# Editorial: Neuroimmune Interface in Health and Diseases

**DOI:** 10.3389/fimmu.2017.01315

**Published:** 2017-10-23

**Authors:** Ihssane Zouikr, Sanae Hasegawa-Ishii, Atsuyoshi Shimada

**Affiliations:** ^1^Laboratory for Molecular Mechanisms of Thalamus Development, RIKEN BSI, Wako, Japan; ^2^Department of Pharmacology, Pennsylvania State University College of Medicine, Hershey, PA, United States; ^3^Faculty of Health Sciences, Kyorin University, Tokyo, Japan

**Keywords:** neuron–glia interactions, amyotrophic lateral sclerosis, pain, BBB transport, mood disorders, neuroinflammation, HIV cure research, bone marrow-derived cells

**Editorial on the Research Topic**

**Neuroimmune Interface in Health and Diseases**

Neuroimmunology is a field that investigates the bi-directional communication between the nervous system (CNS and PNS) and the immune system. While these two physiological systems were traditionally thought to act independently and that the brain was a privileged site protected by the blood–brain barrier (BBB), researchers now appreciate the highly organized cross talk between the immune and nervous systems in health and disease. This conceptual shift came with a series of pioneering experiments by Hugo Besedovsky who demonstrated that the hypothalamic–pituitary–adrenal (HPA) axis and the sympathetic nervous systems were activated in response to a foreign antigen in rats (1). Injection of sheep red blood cells in rats coincides with enhanced levels of circulating corticosterone as well as decreased noradrenaline turn over in the hypothalami of rats. With Charles Dinarello, who first cloned interleukin (IL)-1β, Besedovsky demonstrated that IL-1β is responsible for the development of the brain response to peripheral immune stimulation (2, 3). During the same year, Ed Blalock demonstrated that the production of adrenocorticotropic hormone by leukocytes is induced by corticotropin-releasing hormone (3, 4). The CNS communicates with the immune system *via* hormonal and neural pathways. The hormonal pathway is predominantly *via* the HPA axis, which is the primary stress center in rodents, primates, and humans. The neural pathway is mediated *via* the sympathetic and parasympathetic (the vagus nerve) response. In turn, the immune system signals the CNS *via* cytokines released by activated immune cells in the periphery but also through activated microglia and astrocytes in the spinal cord and brain. The peripheral inflammation can lead to central proinflammatory milieu and ultimately to sickness behavior defined as a set of behavioral changes that develop in individuals during the course of systemic inflammation (i.e., fever, lethargy, hyperalgesia). Cytokines released at the periphery can reach the brain through the circumventricular organs, areas devoid of BBB such as the organum vasculosum lamina terminalis, or through transport and secretion by BBB cells (5).

The access of immune cells and other mediators to the CNS is controlled by the BBB. However, in the event for instance of brain or spinal cord trauma, exposure to environmental toxicants, and inflammation including infection and autoimmune diseases, etc. this barrier can be breached. BBB dysfunction may be seen in a number of neurodegenerative disorders (6) including amyotrophic lateral sclerosis (ALS). Patients with ALS were shown to have enhanced neurovascular permeability (7). However, whether BBB breakdown is the cause or consequence of ALS is still unknown and more studies are needed to clarify the role of BBB breakage in the onset of ALS and of other neurodegenerative disorders.

McKee and Lukens summarize in their review paper the current understanding of the role of immune cells in traumatic brain injury pathogenesis (McKee and Lukens), while Imamura and Hasegawa-Ishii discuss the immune response in the olfactory mucosa following exposure to environmental toxicants (Imamura and Hasegawa-Ishii).

Pain, is the topic of four articles in this special issue. Zouikr and Karshikoff provide an in-depth overview of the role of the cross talk between the endocrine, immune, and central nervous systems in pain as well as the importance of taking early life history into account when treating patients with chronic pain (Zouikr and Karshikoff). Barr and colleagues demonstrate that nerve and root compression in postnatal (P) day 10, 14, 21, and 28 rats produced thermal hyperalgesia and mechanical allodynia that was accompanied by enhanced proinflammatory cytokines and chemokines in the spinal cord. The extent of hyperalgesia and immune activation was greater in older animals (Barr et al.). Pain is a common symptom among patients with multiple sclerosis (MS), using an animal model of MS-induced pain, namely, experimental autoimmune encephalomyelitis (EAE), the team lead by Moalem-Taylor showed that even prior to the onset of clinical EAE, mice already developed mechanical allodynia. This coincided with enhanced levels of Iba1, a marker of microglia, in the spinal cord. Mice with EAE also exhibited increased facial grimacing in the mouse grimace scale during clinical disease (Duffy et al.). Hua in her review argues that opioid-containing immune cells play an important role in peripheral analgesia in inflamed tissue.

Disruption of immune to brain communication is known to increase the susceptibility of developing psychopathological and neurological disorders. In his review, Neupane argues that dysfunctioning of the neuroimmune system could influence the development, progression, and outcome of alcohol use disorder (AUD) and major depression (MD) comorbidity and therefore neuroimmunological alteration should be taken as a key pathophysiological factor when considering the comorbidity between AUD and MD. Karshikoff and Lasselin review the current understanding on the relationship between inflammation and fatigue and argue that the multidimensional aspect of fatigue should be considered when investigating inflammation-induced fatigue (Karshikoff et al.). Edmonson et al. provide a very informative review on the important role of microglia in maintaining a healthy neural network and that abnormal microglial activity can lead to autism. Of particular importance, abnormalities of microglia at the genetic and epigenetic level may contribute to the pathogenesis of autism spectrum disorder. Over-reactivity of microglia has been identified in patients with bipolar disorder, Ohgidani et al. developed a genius technique to induce microglia-like from monocytes. They found a downregulation of CD206, a mannose receptor expressed at the surface of macrophages, endothelial cells, and dendritic cells, during the manic state among three patients with bipolar disorder. This is an important translational study that provides the first evidence that the gene profiling patterns are different between manic and depressive states (Ohgidani et al.). Maintaining a healthy microglia–neuron interaction is critical for neuronal function homeostasis, Wohleb provides an in-depth overview of the importance of neuron–microglia communication in determining homeostatic neuronal function and that alteration in this bi-directional communication can lead to mental health disorders. Microglia can constitute a reservoir for the human immunodeficiency virus (HIV) that use these immune cells to silence its transcription producing a state of viral latency. Marban et al. discuss the different molecular mechanisms involved in the establishment and persistence of HIV latency in brain reservoirs as well as the importance of understanding these molecular mechanisms in order to purge or at least reduce the pool of latently infected brain cells.

The leptomeninges, choroid plexus, attachment of choroid plexus, perivascular space, circumventricular organs, and astrocytic endfeet construct the histological architecture that provides a location for intercellular interactions between bone marrow-derived myeloid lineage cells and brain parenchymal cells under non-inflammatory state but also during the early stages of systemic inflammation. Shimada and Hasegawa-Ishii propose a mechanism connecting systemic inflammation, brain-immune interface cells, and brain parenchymal cells and discuss the relevance of this immune to brain interaction in the context of neurological disorders.

Amyotrophic lateral sclerosis is a debilitating neurodegenerative disorder characterized by a progressive degeneration of motoneurons in the spinal cord and motor cortex. Bone marrow transplantation (BMT) is a promising approach to recompensate the loss of spinal motoneuron in ALS; however, adequate conditioning of BMT is a complex task. In their research article, Peake and colleagues showed that conditioning mice with higher dose of busulfan followed by BMT lead to higher accumulation of bone marrow-derived cells in the spinal cord which was due in part to proliferation of these cells, as well as enhanced microglial activity 7 weeks post-transplant. The authors also demonstrate that in mSOD mice (a mouse model of ALS) conditioned with busulfan, a much higher level of BMDCs accumulation in the spinal cord was observed compared to that of wild-type mice (Peake et al.). However, whether these transplanted stem cells differentiate into motoneurons and whether this is associated with functional recovery in ALS patients is still not known.

As editors, we would like to express our gratitude to all of the scientists around the globe who contributed to this special issue and we hope that you will enjoy reading it.

## Author Contributions

IZ, SH-I, and AS wrote the editorial and approved the final version.

## Conflict of Interest Statement

The authors declare that the research was conducted in the absence of any commercial or financial relationships that could be construed as a potential conflict of interest.
